# Astaxanthin Inhibits ox‐LDL‐Induced Early Endothelial Progenitor Cells Oxidative Stress and Apoptosis Through Nrf2 Activation

**DOI:** 10.1155/sci/1729848

**Published:** 2026-10-26

**Authors:** Keqing Pan, Xiao Xu, Qijun Jiang, Zhigang Gong, Zhigang Li, Shifang Ding, Meichun Zhang

**Affiliations:** ^1^ Wuhan University of Science and Technology School of Medicine Wuhan China; ^2^ Department of Cardiology Wuhan Puren Hospital Wuhan China; ^3^ Department of Cardiology People’s Liberation Army General Hospital of Central Theater Command Wuhan China

**Keywords:** apoptosis, astaxanthin, endothelial progenitor cells, nuclear factor-E2-related factor 2, oxidative stress

## Abstract

Astaxanthin exerts potent protective effects against stem cell apoptosis. This study investigated the protective efficacy of astaxanthin against oxidized low‐density lipoprotein (ox‐LDL)‐induced oxidative stress and apoptosis in endothelial progenitor cells (EPCs). Human early EPCs cultured in vitro were challenged with ox‐LDL in the presence or absence of astaxanthin. Results showed that ox‐LDL exposure significantly reduced nuclear factor‐E2‐related factor 2 (Nrf2) nuclear translocation in EPCs, while increasing oxidative stress and apoptosis rates. Astaxanthin pretreatment attenuated ox‐LDL‐induced reactive oxygen species (ROS) generation and EPC apoptosis, preserved mitochondrial membrane potential (MMP), and concomitantly enhanced nuclear Nrf2 accumulation while upregulating NQO1, HO‐1, and glutathione (GSH) levels. Nrf2 small interfering RNA (siRNA)‐mediated knockdown completely abolished astaxanthin‐induced NQO1 and HO‐1 upregulation. Mechanistically, astaxanthin promoted Akt phosphorylation (activation) and inhibited glycogen synthase kinase 3β (GSK‐3β) phosphorylation. Pretreatment with LY294002 (a phosphoinositide 3‐kinase/PI3K inhibitor) suppressed astaxanthin‐induced Nrf2 nuclear translocation, exacerbated ROS production, and reduced NQO1 and GSH levels. Additionally, astaxanthin blocked ox‐LDL‐induced caspase‐3 upregulation and preserved EPC migration and tube formation. Nrf2 siRNA eliminated the protective effects of astaxanthin on EPC ROS production, MMP preservation, apoptosis, and migration; tube formation was not assessed. Overall, these in vitro findings using cells from healthy donors provide mechanistic support for the potential role of astaxanthin in the PI3K/Akt/GSK‐3/Nrf2 pathway. However, the application of astaxanthin in the prevention and treatment of atherosclerotic disease requires further validation in disease‐relevant models and clinical studies, as the present study was limited to in vitro results.

## 1. Introduction

Despite substantial advances in preventive and therapeutic strategies over the past decades, cardiovascular diseases (CVDs) remain the leading cause of global mortality, accounting for approximately one‐third of all deaths worldwide [1]. Among the multiple pathogenic factors contributing to CVDs, dyslipidemia—particularly elevated plasma low‐density lipoprotein (LDL) and its oxidized derivative (oxidized LDL [ox‐LDL])—plays a pivotal role in the initiation, progression, and destabilization of atherosclerosis, the underlying pathological basis of most CVDs [2]. Accumulating evidence has demonstrated that clinically relevant concentrations of ox‐LDL induce functional impairment and damage in endothelial cells and bone marrow‐derived stem cells, primarily through the excessive generation of reactive oxygen species (ROS) and the subsequent activation of oxidative stress signaling pathways, thereby compromising endothelial repair processes [3].

Endothelial progenitor cells (EPCs), a subset of bone marrow‐derived progenitor cells with the capacity to differentiate into mature endothelial cells, are critical mediators of endothelial regeneration, vascular homeostasis, and postinjury vascular repair [1]. However, exposure of cultured EPCs to ox‐LDL results in dose‐dependent impairment of their key functional properties—including proliferation, migration, and angiogenic capacity—and accelerates cellular senescence and apoptosis [2]. Given the central role of EPC dysfunction in the progression of atherosclerotic CVDs, identifying agents that can protect EPCs against ox‐LDL‐induced injury holds significant therapeutic potential.

Astaxanthin, a naturally occurring non‐provitamin A carotenoid, is highly concentrated in the red pigments of crustaceans (e.g., crabs and shrimp), salmonids (salmon and trout), and asteroidea [3]. Mounting epidemiological and experimental evidence suggests that dietary astaxanthin supplementation reduces the risk of CVDs by targeting multiple pathogenic pathways [4]. Owing to its unique chemical structure—characterized by polar end groups and two oxygenated moieties—astaxanthin exhibits potent ROS‐quenching activity, with antioxidant capacity significantly higher than that of β‐carotene, α‐tocopherol, and other common antioxidants [5]. Beyond direct scavenging of singlet oxygen and free radicals, astaxanthin exerts cytoprotective effects by upregulating the expression of endogenous antioxidant genes, improving mitochondrial function, and inhibiting pro‐inflammatory signaling [6]. Previous studies have shown that astaxanthin protects stem cells and endothelial cells from oxidative stress‐induced dysfunction and apoptosis [7] and contributes to atherosclerosis prevention by inhibiting LDL oxidation, reducing vascular oxidative damage, and limiting lipid deposition in the vascular wall [8].

Nuclear factor‐E2‐related factor 2 (Nrf2) is a key transcription factor that regulates cellular antioxidant and detoxification responses by binding to the antioxidant response element (ARE) in the promoter region of target genes, including NAD(P)H quinone oxidoreductase 1 (NQO1), heme oxygenase‐1 (HO‐1), and glutathione (GSH) synthetase [9–11]. Nrf2 plays a critical role in modulating cellular resistance to oxidative stress and has emerged as a promising therapeutic target in animal models of CVDs [12]. Recent studies have documented high basal expression of Nrf2 in EPCs, where it promotes angiogenesis and maintains EPCs’ functional integrity under physiological conditions [13]. Conversely, ox‐LDL has been reported to inhibit Nrf2 nuclear translocation and downregulate the expression of ARE‐driven antioxidant and oxidative stress response genes in endothelial cells, thereby exacerbating oxidative damage [14]. Notably, astaxanthin has been shown to regulate intracellular ROS levels and exert cytoprotective effects via the activation of the PI3K/Akt and Nrf2/ARE signaling pathways in various cell types [14]. However, whether astaxanthin can protect EPCs against ox‐LDL‐induced injury, and whether this protective effect is mediated by Nrf2 activation via the PI3K/Akt/GSK‐3 pathway, remain incompletely understood.

The present study intended to (1) evaluate the protective role of astaxanthin against ox‐LDL‐mediated oxidative stress, apoptosis, and functional impairment in EPCs and (2) investigate the potential molecular mechanism involving the PI3K/Akt/GSK‐3 pathway and Nrf2 activation. This study might provide new insights into astaxanthin’s development for atherosclerotic CVD prevention and treatment.

## 2. Materials and Methods

### 2.1. Reagents and Materials

Endothelial basal medium (EBM), EGM SingleQuots supplements, and fibronectin were purchased from Lonza (Basel, Switzerland). Fetal bovine serum (FBS) was obtained from Gibco (Thermo Fisher Scientific, Waltham, MA, USA). Astaxanthin (purity ≥ 98%), ox‐LDL, vascular endothelial growth factor (VEGF), Dil‐labeled acetylated LDL (Dil‐ac‐LDL), fluorescein isothiocyanate (FITC)‐conjugated *Ulex europaeus* agglutinin I (UEA‐I), DCFH‐DA, dihydroethidium (DHE), JC‐1, DAPI, and Matrigel were purchased from Sigma–Aldrich (St. Louis, MO, USA). The cell counting kit‐8 (CCK‐8) assay was obtained from Beyotime Institute of Biotechnology (Shanghai, China). Nuclear and Cytoplasmic Protein Extraction Kit was obtained from Applygen Technologies (Beijing, China). Bradford Protein Assay Kit and Enhanced Chemiluminescence (ECL) Detection Kit were purchased from Thermo Fisher Scientific (Waltham, MA, USA). GSH ELISA Kit was obtained from Beyotime Biotechnology (Shanghai, China). Annexin V‐FITC/PI Apoptosis Detection Kit was purchased from BD Biosciences (San Jose, CA, USA). Nrf2 small interfering RNA (siRNA), negative control siRNA, and Lipofectamine 3000 were obtained from Invitrogen (Thermo Fisher Scientific, Waltham, MA, USA). Primary antibodies against Nrf2, Histone H3, β‐actin, NQO1, HO‐1, p‐Akt, Akt, GSK‐3β (Tyr‐216) phosphorylation, GSK‐3β, cleaved caspase‐3, and horseradish peroxidase (HRP)‐conjugated secondary antibodies were purchased from Abcam (Cambridge, UK), Cell Signaling Technology (Danvers, MA, USA), MyBioSource.com (San Diego, California, USA), or BioLegend (San Diego, CA, USA). Primary antibodies against CD34, CD133, and VEGFR2/KDR were purchased from BioLegend (San Diego, CA, USA). The TRIzol reagent and SYBR‐Green qPCR Kit were obtained from Ambion (Thermo Fisher Scientific, Waltham, MA, USA). The 96‐well/6‐well culture plates were purchased from Corning (Corning, NY, USA). Transwell chambers (8 μm pore size) were purchased from Corning (Millipore, Billerica, MA, USA).

### 2.2. Isolation and Culture of Human Early EPCs

Peripheral blood mononuclear cells (PBMCs) were isolated from 20 mL of fresh peripheral blood using Ficoll‐Paque density gradient centrifugation (1800 rpm, 20 min, room temperature). Isolated PBMCs were washed twice with phosphate‐buffered saline (PBS) and resuspended in EBM supplemented with EGM SingleQuots (containing insulin‐like growth factor‐1, basic fibroblast growth factor, epidermal growth factor, and hydrocortisone), VEGF (10 ng/mL), and 20% FBS at a density of 5 × 10^6^ cells/mL. The cell suspension was plated onto fibronectin‐coated (10 μg/cm^2^) 6‐well culture plates and incubated at 37°C in a humidified atmosphere with 5% CO_2_. Nonadherent cells were removed by replacing the medium after 48 h, and the culture medium was refreshed every 2 days thereafter. After 7 days of primary culture, adherent cells (EPCs) were harvested with 0.25% trypsin‐EDTA, passaged at a ratio of 1:2, and cultured until 80% confluency. Prior to experimental interventions, early EPCs were serum‐starved in EBM containing 0.5% FBS for 12 h to synchronize the cell cycle.

### 2.3. Early EPCs Phenotypic Identification

Morphological observation: early EPCs morphology was monitored daily using an inverted phase‐contrast microscope (Olympus, Tokyo, Japan), and images were captured on day 7 of culture.

Dil‐ac‐LDL uptake and UEA‐I binding assay: early EPCs were seeded onto fibronectin‐coated glass coverslips in 24‐well plates. After 24 h, cells were incubated with Dil‐ac‐LDL (10 μg/mL) at 37°C for 4 h, washed three times with PBS, and then incubated with FITC‐UEA‐I (10 μg/mL) at 37°C for 1 h. After washing with PBS, cells were fixed with 4% paraformaldehyde for 15 min at room temperature. Fluorescent images were captured using an inverted fluorescence microscope (Olympus, Tokyo, Japan), and the percentage of double‐positive cells (Dil‐ac‐LDL^+^/UEA‐I^+^) was calculated by counting ≥5 random fields per coverslip (≥200 cells/field).

For flow cytometry, cells were harvested with trypsin‐EDTA, washed twice with PBS, and incubated with CD34, CD133, and VEGFR2/KDR primary antibodies for 15 min at room temperature in the dark, and were analyzed by flow cytometry within 1 h. Single cells were gated after exclusion of debris and cellular aggregates on an FSC‐A vs. SSC‐A scatter plot, and then CD34+ cells were gated within this population on a CD34 vs. SSC‐A plot The expression of CD34 and CD133 was assessed in all cells, while VEGFR2/KDR expression was assessed in CD34+ cells. Irrelevant antibodies matched for host species, isotype, and fluorochrome conjugation (e.g., IgG1‐FITC and IgG1‐PE) were used as isotype controls to assess nonspecific binding for each marker.

### 2.4. Experimental Grouping and Study Design

Control group: early EPCs cultured in a complete medium without any treatment. ox‐LDL group: early EPCs treated with 20 μg/mL ox‐LDL for 24 h (unless otherwise specified). Astaxanthin pretreatment groups: early EPCs pretreated with astaxanthin (10, 50, or 100 μg/mL) for 6 h prior to ox‐LDL (20 μg/mL) exposure for 24 h. Nrf2 siRNA group: early EPCs transfected with Nrf2 siRNA (50 nM) for 48 h, followed by astaxanthin (50 μg/mL) pretreatment for 6 h. Negative control siRNA group: early EPCs transfected with negative control siRNA (50 nM) under the same conditions as the Nrf2 siRNA group. LY294002 group: early EPCs pretreated with the PI3K inhibitor LY294002 (1 μM) for 30 min, followed by astaxanthin (50 μg/mL) pretreatment for 6 h. Astaxanthin was dissolved in dimethyl sulfoxide (DMSO) to prepare a stock solution (100 mg/mL), and the final DMSO concentration in the culture medium was ≤0.1% (v/v), which had no significant effect on early EPCs viability.

### 2.5. Cell Viability Assay

The viability of early EPCs was determined using the CCK‐8 assay (Beyotime Institute of Biotechnology, Shanghai, China) according to the manufacturer’s instructions. Early EPCs were cultured in 96‐well plates (seeding density: 1 × 103 cells/100 μL) and incubated with various ways of intervention. Early EPCs were washed with PBS and incubated with CCK‐8 solution (1:10 dilution) for 1 h and then measured with a microplate reader at the absorbance 450 nm.

### 2.6. Detection of Intracellular ROS Levels

Intracellular ROS levels were detected using a DCFH‐DA (general ROS probe). Early EPCs were seeded in 6‐well plates (5 × 10^5^ cells/well) and treated as described above. After treatment, the cells were incubated with DCFH‐DA (10 μM) at 37°C for 30 min in the dark. For fluorescence microscopy, cells were washed three times with PBS, and images were captured using an inverted fluorescence microscope (Olympus, Tokyo, Japan) with the same exposure parameters (excited at 488 nm and received at 525 nm). Fluorescence images were captured, and the mean fluorescence intensity of the early EPCs was analyzed using Image‐Pro Plus 6.0 software (Media Cybernetics, Rockville, MD, USA). Each cell group was measured six times. All measurements were performed by three independent researchers.

For flow cytometry, cells were harvested with trypsin‐EDTA, washed twice with PBS, and analyzed using a BD FACSCanto II flow cytometer (BD Biosciences, San Jose, CA, USA) with the same parameters as the FITC channel (488 nm excitation, 525 nm reception). ROS levels were evaluated by calculating the proportion of cells with a high fluorescence intensity.

### 2.7. Measurement of Mitochondrial Membrane Potential (MMP; ΔΨm)

MMP was assessed using the JC‐1 assay. Early EPCs were seeded in 6‐well plates (5 × 10^5^ cells/well) and treated as indicated. After treatment, the cells were incubated with JC‐1 working solution (5 μM) at 37°C for 20 min in the dark. Cells were then washed twice with JC‐1 buffer, and fluorescence images were captured using an inverted fluorescence microscope (Olympus, Tokyo, Japan) with excitation wavelengths of 488 nm (green fluorescence) and 550 nm (red fluorescence). For quantitative analysis, cells were harvested, resuspended in JC‐1 buffer, and analyzed by flow cytometry. The ΔΨm was represented as the ratio of red fluorescence intensity (JC‐1 aggregates) to green fluorescence intensity (JC‐1 monomers). MMP (ΔΨm) was evaluated using, where intact mitochondria (high ΔΨm) form red fluorescent aggregates, and depolarized mitochondria emit green fluorescent monomers.

### 2.8. Determination of Intracellular GSH Levels

Intracellular GSH levels were measured using a GSH ELISA Kit according to the manufacturer’s instructions. Early EPCs were seeded in 96‐well plates (1 × 10^4^ cells/well) and treated as described. After treatment, cells were lysed with ice‐cold lysis buffer, and the cell lysate was centrifuged at 12,000 rpm for 10 min at 4°C to collect the supernatant. The supernatant was mixed with reaction buffer and chromogenic substrate and incubated at 37°C for 30 min, and the absorbance was measured at 405 nm using a microplate reader (Thermo Fisher Scientific, Waltham, MA, USA). GSH concentrations were calculated based on a standard curve and normalized to the total protein content of the supernatant (determined by the Bradford assay).

### 2.9. Apoptosis Assay

DAPI staining: early EPCs were seeded on fibronectin‐coated glass coverslips in 24‐well plates (2 × 10^4^ cells/well) and treated as indicated. After treatment, the cells were fixed with 4% paraformaldehyde for 15 min, washed with PBS, and stained with DAPI (5 μg/mL) for 5 min. Fluorescent images were captured using a fluorescence microscope, and the percentage of apoptotic cells was determined by counting the number of cells with pyknotic, condensed nuclei among ≥200 cells per random field (≥5 fields/coverslip).

Annexin V‐FITC/PI double staining: early EPCs were seeded in 6‐well plates (5 × 10^5^ cells/well) and treated as described. After treatment, cells were harvested with trypsin‐EDTA, washed twice with cold PBS, and resuspended in binding buffer at a density of 1 × 10^6^ cells/mL. Cells were incubated with Annexin V‐FITC (5 μL) and PI (10 μL) for 15 min at room temperature in the dark, and apoptotic rates were analyzed by flow cytometry within 1 h. The total apoptotic rate was defined as the percentage of Annexin V‐positive cells (early apoptosis: Annexin V^+^/PI^−^; late apoptosis: Annexin V^+^/PI^+^).

### 2.10. Cytoplasmic and Nuclear Protein Extraction and Western Blot Analysis

Early EPCs were seeded in 6‐well plates (1 × 10^6^ cells/well) and treated as indicated. Cytoplasmic and nuclear proteins were extracted using a Nuclear and Cytoplasmic Protein Extraction Kit according to the manufacturer’s protocol. Cells were washed twice with ice‐cold pPBS, scraped from culture dishes on ice using a plastic cell scraper, and collected in 1.5 mL microcentrifuge tubes in 1 mL of ice‐cold PBS. After centrifugation for 10 s and removal of supernatants, precipitations were resuspended in 600 μL of ice‐cold cytoplasmic extraction buffer, vortexed vigorously for 30 s, incubated on ice for 15 min, and centrifuged at 4°C 16,000 g for 5 min. The supernatants were completely and carefully removed as the “cytosolic fraction.” About 300 μL of nuclear extraction buffer was added to the precipitations, vortexed vigorously for 30 s, and incubated on ice for 1 min. Repeat vortexing for 30 s and incubation for 1 min four times. The liquid was designated as the “nuclear fraction.” A 100 μL sample was used to determine the protein concentration by the Bradford assay. Protein samples (30 μg per lane) of equal loading were resolved using 12% sodium dodecyl sulfate‐polyacrylamide gel electrophoresis (SDS‐PAGE) and then blotted onto nitrocellulose membranes (Millipore, Billerica, MA, USA). Membranes were blocked for 1 h at room temperature using 5% nonfat milk prepared in TBS/T, and then incubated overnight with primary antibodies at a dilution of 1:1000 at 4°C. After washing three times with TBS/T (10 min each), membranes were incubated with HRP‐conjugated secondary antibodies (1:5000 dilution) for 1 h at room temperature. Protein bands were visualized using an ECL Detection Kit, and images were captured using a ChemiDoc XRS + Imaging System (Bio‐Rad, Hercules, CA, USA). Band intensities were quantified using Image‐Pro Plus 6.0 software (Media Cybernetics, Rockville, MD, USA) and normalized to the internal reference proteins (β‐actin for cytoplasmic proteins and Histone H3 for nuclear proteins). The fractionation of nuclear/cytoplasmic was validated by cross‐detection of internal reference proteins.

### 2.11. RNA Extraction and RT‐PCR Analysis

Total RNA was extracted from early EPCs using the Trizol reagent according to the manufacturer’s instructions. RNA purity and concentration were determined by measuring the absorbance ratio at 260/280 nm (1.8–2.0) using a NanoDrop 2000 spectrophotometer (Thermo Fisher Scientific, Waltham, MA, USA). Complementary DNA (cDNA) was synthesized from 1 μg of total RNA using a reverse transcription kit (Takara, Dalian, China). Quantitative real‐time PCR (qPCR) was performed using a SYBR‐Green qPCR Kit on a StepOnePlus Real‐Time PCR System (Thermo Fisher Scientific, Waltham, MA, USA). The primer sequences were as follows:

NQO1: forward 5′‐CGCAGACCTTGTGATA‐3′, reverse 5′‐TGGCAGCGTAAGTGTA‐3′
HO‐1: forward 5′‐TTCAAGCAGCTCTACCGCTC‐3′, reverse 5′‐GAACGCAGTCTTGGCCTCTT‐3′
GAPDH: forward 5′‐ACAACTTTGGTATCGTGGAAGG‐3′, reverse 5′‐GCCATC ACGCCA CAG TTT C‐3′ (internal reference gene)

The qPCR thermal cycling parameters were as follows: 30 s at 95°C, then 40 cycles consisting of 5 s at 95°C, and 30 s at 60°C. The relative mRNA expression levels were calculated using the 2^−ΔΔCt^ method and normalized to GAPDH.

### 2.12. siRNA Transfection

Early EPCs were seeded in 6‐well plates (5 × 10^5^ cells/well) and cultured until 50%–60% confluency. Nrf2 siRNA or negative control siRNA (50 nM) was transfected into early EPCs using Lipofectamine 3000 according to the manufacturer’s protocol. Briefly, siRNA and Lipofectamine 3000 were diluted in Opti‐MEM medium (Invitrogen, Thermo Fisher Scientific, Waltham, MA, USA) separately, incubated at room temperature for 5 min, then mixed, and incubated for another 20 min. The mixture was added to the culture medium, and cells were incubated at 37°C for 48 h. The efficiency of Nrf2 knockdown was verified by western blot analysis.

### 2.13. Transwell Migration Assay

Early EPC migration was assessed using Transwell chambers with an 8 μm pore size. Early EPCs were treated as indicated, harvested with trypsin‐EDTA, and resuspended in serum‐free EBM at a density of 5 × 10^4^ cells/100 μL. The cell suspension was added to the upper chamber, and 600 μL of complete EBM (containing 20% FBS and 10 ng/mL VEGF) was added to the lower chamber as a chemoattractant. Cells were incubated at 37°C for 24 h, and then nonmigrated cells on the upper surface of the membrane were removed with a cotton swab. The migrated cells on the lower surface were fixed with 4% paraformaldehyde for 15 min, stained with 0.1% crystal violet for 10 min, and washed with PBS. Images were captured using an inverted microscope (Olympus, Tokyo, Japan), and the number of migrated cells was counted in ≥5 random fields per chamber (200× magnification).

### 2.14. Tube Formation Assay

Early EPCs angiogenic capacity was evaluated using a Matrigel tube formation assay. Matrigel was thawed overnight at 4°C, added to 96‐well plates (50 μL/well), and polymerized at 37°C for 30 min. Early EPCs were treated as indicated, harvested, and resuspended in complete EBM at a density of 2 × 10^4^ cells/100 μL. The cell suspension was added to the Matrigel‐coated wells and incubated at 37°C for 6 h. Tube formation was observed using an inverted phase‐contrast microscope, and images were captured. The total tube length and number of tube branches were quantified using ImageJ software (National Institutes of Health, Bethesda, MD, USA) in ≥5 random fields per well.

### 2.15. Statistical Analysis

All experiments were performed in triplicate and repeated at least three times independently. Results are shown as the mean ± SD. Statistical analysis was performed using SPSS 22.0 software (IBM, Armonk, NY, USA). Differences between multiple groups were analyzed by one‐way analysis of variance (ANOVA) followed by Tukey’s post hoc test. *p*  < 0.05 was considered statistically significant.

## 3. Results

### 3.1. Early EPC Morphology and Phenotypic Identification

Human early EPCs were isolated from PBMCs of healthy volunteers (eight independent healthy volunteers, mean age 31.3 years; 3 males and 5 females). Cells adherent to the fibronectin‐coated culture dishes 24 h after the seeding, initial colony formation emerged at 48 h, and the robust colony expanded by day 4 of culture (Figure 1A). Consistent with the established morphological features of early EPCs, adherent cells exhibited two characteristic morphological phenotypes: cobblestone‐like (endothelial‐like) and spindle‐shaped (fibroblast‐like) after 7 days of in vitro culture (Figure 1B,C). Most adherent cells uptook Dil‐labeled acetylated LDL (Dil‐ac‐LDL, red fluorescence, Figure 1B) efficiently, bound to FITC‐conjugated UEA‐I (green fluorescence, Figure 1C), and were double‐positive for both markers as merged (Figure 1D). More than 90% of the positive proportion of CD34, CD133, and VEGFR2/KDR confirmed early EPCs phenotype (Figure 1E).

**Figure 1:**
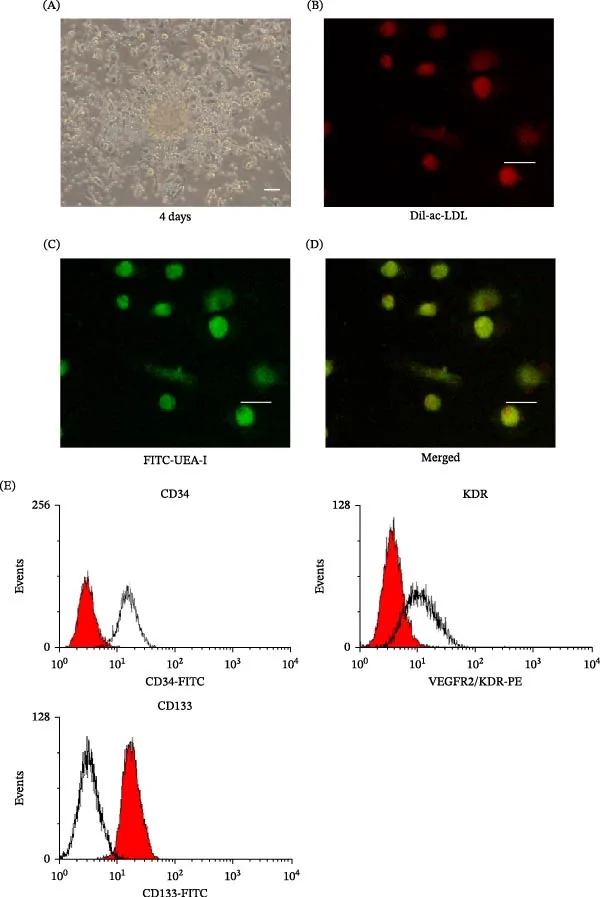
Morphological characteristics and phenotypic identification of human early EPCs. (A–D) After 7 days in vitro culture of PBMCs, adherent cells exhibited spindle‐shaped (fibroblast‐like) and cobblestone‐like (endothelial‐like) phenotypes (scale bar: 100 μm). Over 90% of cells were double‐positive for Dil‐ac‐LDL (red fluorescence, B) and UEA‐I (green fluorescence, C), as the merged images (D), (scale bar: 50 μm). Most cells were positive for CD34, CD133, and VEGFR2/KDR by flow cytometry detection (E). The positivity rates for CD34, VEGFR2/KDR, and CD133 were (95.5 ± 2.4)%, (90.7 ± 2.8)%, and (93.4 ± 1.8)%, respectively (mean ± SD). Experiments were performed using samples from three independent donors (*n* = 3). For each donor, technical replicates were performed in triplicate. The average positive proportion for each marker was calculated for each donor. Results are expressed as donor‐level mean ± SD.

### 3.2. Astaxanthin Attenuates ox‐LDL‐Induced Oxidative Stress in Early EPCs

Our previous study has reported that challenging EPCs with 20 μg/mL ox‐LDL for 24 h induced significant oxidative stress in EPCs [15]. This study showed that treatment with 20 μg/mL ox‐LDL for 24 h impaired early EPCs viability, but 10, 50, or 100 μg/mL astaxanthin did not (Figure S1).

To evaluate the effect of astaxanthin on ox‐LDL‐induced oxidative damage, ROS production, MMP, and GSH levels were assessed after the treatment of 10, 50, and 100 μg/mL astaxanthin for 6 h and 20 μg/mL ox‐LDL exposure for 24 h. Fluorescence microscopy and flow cytometry analyses with DCFH‐DA (a general ROS probe) and DHE (a superoxide‐specific probe) consistently showed that intracellular ROS levels significantly increased after ox‐LDL treatment and were significantly inhibited by pretreatment with 10, 50, or 100 μg/mL astaxanthin (Figure 2A–C; Control groups, Figures S2 and S3). However, the effects of astaxanthin on ROS production could be reversed by Nrf2 siRNA intervention (Figure 2A–C).

Figure 2Astaxanthin attenuates ox‐LDL‐induced oxidative stress in early EPCs. Early EPCs were pretreated with astaxanthin (10, 50, or 100 μg/mL) for 6 h, followed by exposure to 20 μg/mL ox‐LDL for 24 h. (A–C) Intracellular ROS levels detected by DCFH‐DA and DHE staining (A, scale bar: 100 μm, B), with quantitative analysis by flow cytometry (C). Astaxanthin dose‐dependently reduced ox‐LDL‐induced ROS elevation. (D, E) Mitochondrial membrane potential (ΔΨm) assessed by JC‐1 staining. ox‐LDL‐induced mitochondrial depolarization (increased green fluorescence) was reversed by astaxanthin, as indicated by the increased red/green fluorescence ratio (D, scale bar: 50 μm, E). (F) Intracellular GSH levels measured by ELISA. ox‐LDL (10 or 20 μg/mL) reduced GSH levels, which was abrogated by 50 μg/mL astaxanthin pretreatment. Nrf2 siRNA treatment reversed the effects of astaxanthin on ROS production (A–C), mitochondrial membrane potential (D, E) and intracellular GSH levels (F). Experiments were performed using samples from six independent donors (*n* = 6). For each donor, detection was technically replicated six times, and three image fields were captured per technical replicate. The relative mean fluorescence intensity normalized to the control group was calculated for each donor and expressed as mean ± SD in ROS experiments. The relative mean red/green fluorescence ratio normalized to the control group was calculated for each donor and expressed as mean ± SD in mitochondrial membrane potential experiments. The relative GSH levels normalized to the control group were calculated for each donor and expressed as mean ± SD in GSH experiments. These donor‐level values were then used for statistical comparison among groups. Comparisons among multiple groups were performed using one‐way ANOVA followed by Tukey’s post hoc test in SPSS 22.0.  ^∗^ *p* < 0.01 vs. control;  ^∗∗^ *p* < 0.01 vs. ox‐LDL group; #*p* < 0.01 vs. ox‐LDL + 50 μg/mL astaxanthin group. Ast, astaxanthin.

**Figure:**
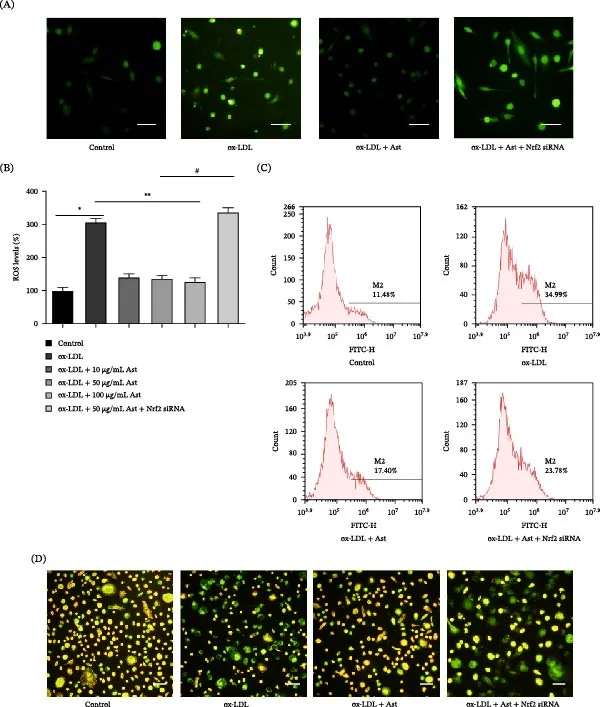

**Figure:**
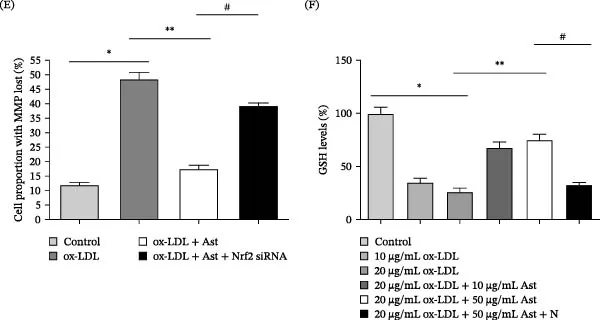

JC‐1 staining showed ox‐LDL treatment markedly increased green fluorescence intensity (*p* < 0.01), indicating severe mitochondrial depolarization (low ΔΨm). However, pretreatment with astaxanthin before ox‐LDL exposure significantly increased in the red/green fluorescence ratio (Figure 2D,E; control groups, Figure S4), indicating preservation of mitochondrial membrane integrity. The effects of astaxanthin on MMP could be reversed by Nrf2 siRNA intervention (Figure 2D,E).

ELISA‐based quantification of intracellular GSH levels revealed that ox‐LDL treatment (10 or 20 μg/mL) significantly reduced the GSH content compared to the control group (Figure 2F). Pretreatment with 10 and 50 μg/mL astaxanthin significantly abrogated the ox‐LDL‐induced GSH reduction (Figure 2F). The effects of astaxanthin on GSH production could be reversed by Nrf2 siRNA pretreatment (Figure 2F). Collectively, these data demonstrate that astaxanthin effectively mitigates ox‐LDL‐induced oxidative stress in early EPCs by reducing ROS production, preserving mitochondrial function, and restoring endogenous antioxidant GSH levels.

### 3.3. Astaxanthin Suppresses ox‐LDL‐Induced Early EPCs Apoptosis

Early EPCs were pretreated with 10 μg/mL astaxanthin for 6 h, followed by 20 μg/mL ox‐LDL treatment for 24 h. DAPI staining showed ox‐LDL (20 μg/mL) treatment for 24 h resulted in obvious morphological signs of apoptosis, including cell shrinkage and nuclear pyknosis, also rises of percentage of pyknotic nuclei (ox‐LDL group vs. control group: 13.5 ± 1.3% vs. 4.1 ± 0.5%, *p* < 0.01; Figure 3A, C; control groups, Figure S5). Pretreatment with 10 μg/mL astaxanthin reversed the effects of ox‐LDL on early EPCs apoptosis, and reduced the percentage of pyknotic nuclei to 6.7 ± 0.5% (*p* < 0.01 vs. ox‐LDL alone group; Figure 3A,C).

**Figure 3:**
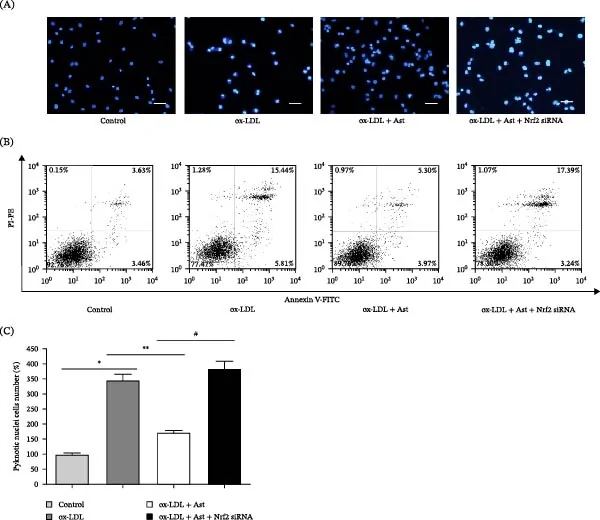
Astaxanthin suppresses ox‐LDL‐induced early EPCs apoptosis. Early EPCs were pretreated with 10 μg/mL astaxanthin for 6 h, followed by 20 μg/mL ox‐LDL treatment for 24 h. (A, C) Apoptotic morphology detected by DAPI staining. ox‐LDL increased cell shrinkage and nuclear pyknosis (A, scale bar: 50 μm), while astaxanthin reduced the percentage of cell shrinkage and pyknotic nuclei (A, C). Quantitative analyses are performed by three independent experimenters. (B) Apoptotic rates analyzed by Annexin V‐FITC/PI double staining and flow cytometry. Astaxanthin significantly reduced ox‐LDL‐induced total apoptotic rate (Annexin V‐FITC/PI‐double positive cells). Nrf2 siRNA treatment reversed the effects of astaxanthin on apoptosis (A–C). DAPI staining experiments were performed using samples from six independent donors (*n* = 6). For each donor, DAPI staining was technically replicated six times, and three image fields were captured per technical replicate. The numbers of cells with pyknotic, condensed nuclei normalized to the control group were calculated for each donor and expressed as mean ± SD. These donor‐level values were then used for statistical comparison among groups. Comparisons among multiple groups were performed using one‐way analysis of variance (ANOVA) followed by Tukey’s post hoc test, with SPSS 22.0.  ^∗^ *p* < 0.01 vs. control;  ^∗∗^ *p* < 0.01 vs. ox‐LDL group; #*p* < 0.01 vs. ox‐LDL + astaxanthin group. Ast, astaxanthin.

Flow cytometry analysis of Annexin V/PI‐stained cells showed that ox‐LDL treatment increased the total apoptotic rate (Annexin V‐FITC/PI‐double positive cells) from 3.8 ± 0.8% (control) to 16.2 ± 2.4% (ox‐LDL alone, *p* < 0.01; Figure 3B). Pretreatment with 10 μg/mL astaxanthin significantly reduced the apoptotic rate to 5.7 ± 1.6% (*p* < 0.01 vs. ox‐LDL alone; Figure 3B; Astaxanthin control group, Figure S6). However, the effects of astaxanthin on apoptosis were reversed by pretreatment of Nrf2 siRNA (Figure 3B). These results collectively confirm that astaxanthin exerts a potent antiapoptotic effect against ox‐LDL‐induced early EPCs injury.

### 3.4. Astaxanthin Promotes Nrf2 Nuclear Translocation in Early EPCs

The distribution of nuclear factor‐E2‐related factor 2 (Nrf2) in cytoplasmic and nuclear fractions was tested with western blot for the evaluation of Nrf2 activation. Result showed that treatment with ox‐LDL (20 μg/mL) for 12 h significantly reduced nuclear Nrf2 protein levels (0.44 ± 0.05‐fold vs. control, *p* < 0.01; Figures 4A,B and S8) without affecting total Nrf2 mRNA expression (data not shown), indicating impaired Nrf2 nuclear translocation. Pretreatment with astaxanthin (10 or 50 μg/mL) prior to ox‐LDL exposure markedly increased nuclear Nrf2 accumulation (1.91 ± 0.18‐fold and 2.69 ± 0.23‐fold vs. ox‐LDL alone group, respectively; *p* < 0.01 for both; Figures 4A,B and S8). The ratio of nuclear Nrf2 (normalized to Histone H3) to cytoplasmic Nrf2 (normalized to β‐actin) was significantly higher in astaxanthin‐pretreated groups (*p* < 0.01 vs. ox‐LDL alone; Figures 4B and S8), confirming that astaxanthin promotes Nrf2 nuclear translocation rather than regulating its transcriptional synthesis or total protein expression.

**Figure 4:**
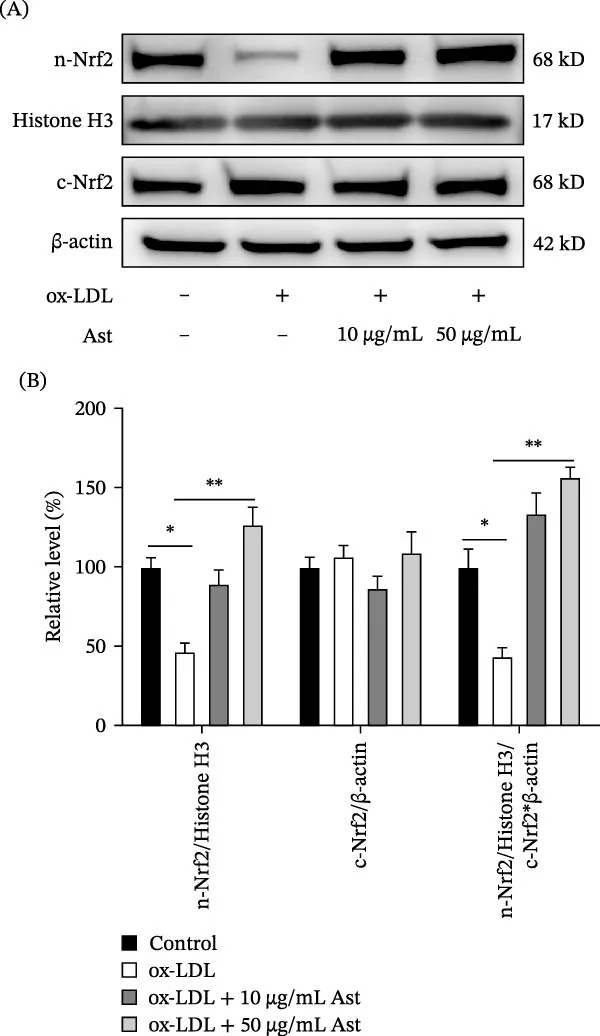
Astaxanthin promotes Nrf2 nuclear translocation in early EPCs. Early EPCs were pretreated with astaxanthin (10 or 50 μg/mL) for 6 h, followed by 20 μg/mL ox‐LDL treatment for 12 h. (A) Representative western blot images of cytoplasmic and nuclear Nrf2 protein levels. β‐actin and Histone H3 were used as internal references for cytoplasmic and nuclear proteins, respectively. (B) Quantitative analysis of the nuclear/cytoplasmic Nrf2 ratio. ox‐LDL reduced nuclear Nrf2 accumulation, while astaxanthin significantly enhanced Nrf2 nuclear translocation. Nrf2 nuclear translocation experiments were performed using samples from six independent donors (*n* = 6). For each donor, western blot analysis was technically replicated three times. The relative Nrf2 levels and the nuclear/cytoplasmic Nrf2 ratio normalized to the control group were calculated for each donor and expressed as mean ± SD. These donor‐level values were then used for statistical comparison among groups. Comparisons among multiple groups were performed using one‐way analysis of variance (ANOVA) followed by Tukey’s post hoc test, with SPSS 22.0.  ^∗^ *p* < 0.01 vs. control;  ^∗∗^ *p* < 0.01 vs. ox‐LDL group. Ast, astaxanthin.

### 3.5. Astaxanthin Upregulates NQO1 and HO‐1 Expression via Nrf2‐Dependent Mechanism

The protein and mRNA levels of Nrf2 downstream target genes, NAD(P)H quinone oxidoreductase 1 (NQO1) and heme oxygenase‐1 (HO‐1) are tested with western blot and RT‐PCR, respectively. Treatment of 20 μg/mL ox‐LDL for 24 h significantly decreased the protein levels of NQO1 and HO‐1 (Figures 5A–C and S9). However, pretreatment of 50 μg/mL astaxanthin significantly reversed ox‐LDL‐induced depression of NQO1 and HO‐1 protein expression levels (Figures 5A–C and S9).

**Figure 5:**
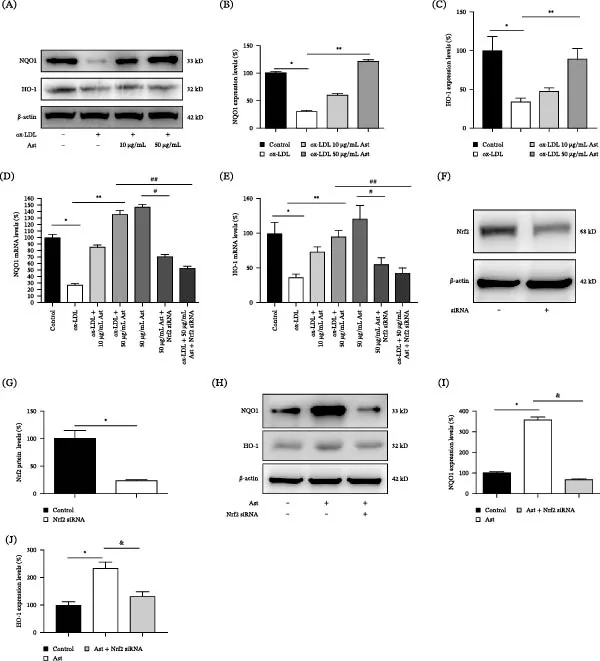
Astaxanthin upregulates NQO1 and HO‐1 expression via Nrf2‐dependent mechanism. (A–C) Early EPCs were pretreated with astaxanthin (10 or 50 μg/mL) for 6 h, followed by 20 μg/mL ox‐LDL treatment for 24 h. (A–C) NQO1 and HO‐1 protein levels detected by western blot. NQO1 (D) and HO‐1 (E) mRNA levels measured by RT‐PCR. Astaxanthin dose‐dependently reversed ox‐LDL‐induced downregulation of NQO1 and HO‐1 mRNA and protein. (D–J) Early EPCs were transfected with Nrf2 siRNA or negative control siRNA for 48 h, followed by 50 μg/mL astaxanthin pretreatment and 20 μg/mL ox‐LDL treatment. (F, G) Nrf2 protein levels after siRNA transfection. Nrf2 siRNA knockdown abolished astaxanthin‐induced upregulation of NQO1 and HO‐1 mRNA (D, E) and protein levels (H–J). Experiments were performed using samples from six independent donors (*n* = 6). For each donor, western blot and RT‐PCR analysis were technically replicated three times. For each donor, NQO1, HO‐1, and Nrf2 protein levels and NQO1 and HO‐1 mRNA levels were normalized to the control group and expressed as relative levels (mean ± SD). These donor‐level values were then used for statistical comparison among groups. Comparisons among multiple groups were performed using one‐way analysis of variance (ANOVA) followed by Tukey’s post hoc test, with SPSS 22.0.  ^∗^ *p* < 0.01 vs. control group;  ^∗∗^ *p* < 0.01 vs. ox‐LDL alone group; #*p* < 0.01 vs. astaxanthin group; ##*p* < 0.01 vs. ox‐LDL + 50μg/mL astaxanthin group; &*p* < 0.01 vs. astaxanthin alone group. Ast, astaxanthin.

Consistent with western blot results, treatment of ox‐LDL (20 μg/mL) for 24 h significantly downregulated mRNA expression of NQO1 (0.27 ± 0.03‐fold vs. control, *p* < 0.01; Figure 5D) and HO‐1 (0.36 ± 0.04‐fold vs. control, *p* < 0.01; Figure 5E). Pretreatment with astaxanthin (10 or 50 μg/mL) dose‐dependently increased mRNA levels of NQO1 (10 μg/mL astaxanthin 0.86 ± 0.05‐fold and 50 μg/mL astaxanthin 1.36 ± 0.10‐fold vs. control group respectively; *p* < 0.01, both; Figure 5D) and HO‐1 (10 μg/mL astaxanthin 0.74 ± 0.05‐fold and 50 μg/mL astaxanthin 0.95 ± 0.06‐fold vs. control group respectively; *p* < 0.01, both; Figure 5E).

Nrf2 siRNA were synthesized and transfected into early EPCs to determine the effects of Nrf2 on astaxanthin‐induced NQO1 and HO‐1 expression. Nrf2 protein levels are efficiently knocked down by Nrf2 siRNA (0.24 ± 0.06‐fold vs. negative control siRNA, *p* < 0.01; Figures 5F,G and S10). Astaxanthin‐induced expression of NQO1 and HO‐1 mRNA (Figure 5D,E) and protein (Figures 5H–J and S11) were completely abolished by Nrf2 knockdown, demonstrating the dependence of astaxanthin‐induced NQO1 and HO‐1 expression on Nrf2.

### 3.6. Astaxanthin Activates Nrf2 via the PI3K/Akt/GSK‐3 Pathway

The upstream of the Nrf2 signal, Akt and glycogen synthase kinase 3 (GSK‐3) were tested with western blot after astaxanthin intervention. Treatment with astaxanthin (10 or 50 μg/mL) for 6 h significantly increased Akt phosphorylation (p‐Akt/Akt ratio: 3.23 ± 0.21 and 3.85 ± 0.10‐fold vs. control, respectively; *p* < 0.01; Figures 6A,B and S12) and inhibited GSK‐3β (Tyr‐216) phosphorylation (p‐GSK‐3/GSK‐3 ratio: 0.38 ± 0.04 and 0.32 ± 0.05‐fold vs. control, respectively; *p* < 0.01; Figures 6C,D and S13), indicating activation of the PI3K/Akt pathway and inhibition of GSK‐3 activity.

**Figure 6:**
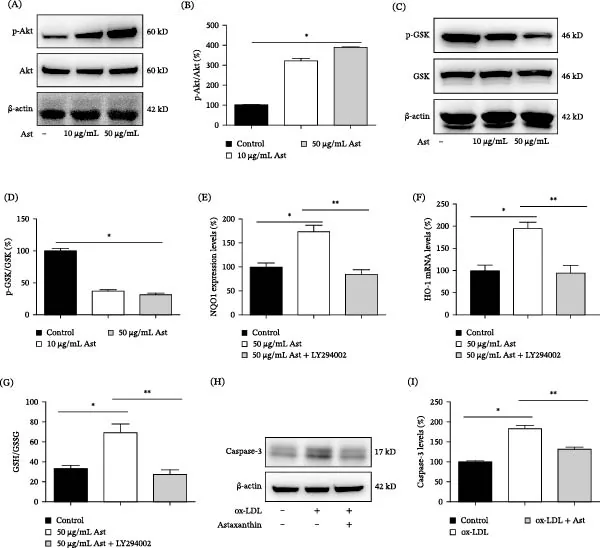
Astaxanthin activates Nrf2 via the PI3K/Akt/GSK‐3 pathway and inhibits caspase‐3 activation. (A–D) Early EPCs were treated with astaxanthin (10 or 50 μg/mL) for 6 h. (A, C) Representative western blot images of p‐Akt, Akt, p‐GSK‐3, and GSK‐3 protein levels. (B, D) Quantitative analysis of p‐Akt/Akt and p‐GSK‐3/GSK‐3 ratios. Astaxanthin enhanced Akt phosphorylation and inhibited GSK‐3β (Tyr‐216) phosphorylation. (E–G) Early EPCs were pretreated with 1 μM LY294002 for 30 min, followed by 50 μg/mL astaxanthin. LY294002 abolished astaxanthin‐induced NQO1 (E) and HO‐1 (F) mRNA upregulation and GSH production (G). (H, I) Early EPCs were pretreated with 50 μg/mL astaxanthin for 6 h, followed by 20 μg/mL ox‐LDL treatment for 24 h. (H) Representative western blot images of cleaved caspase‐3 protein levels. (I) Quantitative analysis. Astaxanthin blocked ox‐LDL‐induced cleaved caspase‐3 upregulation. Experiments were performed using samples from five independent donors (*n* = 5). For each donor, detection of p‐Akt, Akt, p‐GSK‐3, GSK‐3, cleaved caspase‐3, and β‐actin was technically replicated three times, and detection of NQO1 and HO‐1 mRNA and GSH was technically replicated six times. The relative p‐Akt/Akt, p‐GSK‐3/GSK‐3, cleaved caspase‐3 levels, relative NQO1 and HO‐1 mRNA levels and GSH levels normalized to the control group were calculated for each donor and expressed as mean ± SD. These donor‐level values were then used for statistical comparison among groups. Comparisons among multiple groups were performed using one‐way analysis of variance (ANOVA) followed by Tukey’s post hoc test, with SPSS 22.0.  ^∗^ *p* < 0.01 vs. control group;  ^∗∗^ *p* < 0.01 vs. ox‐LDL alone group. Ast, astaxanthin.

The NQO1 mRNA and GSH production were tested after the pretreatment of the specific PI3K inhibitor LY294002 (1 μM) for 30 min and intervention of astaxanthin. LY294002 pretreatment before astaxanthin intervention significantly decreased NQO1 and HO‐1 mRNA levels (Figure 6E,F) and GSH production (Figure 6G). These results confirm that astaxanthin activates Nrf2 signaling primarily through the PI3K/Akt/GSK‐3 pathway in early EPCs.

### 3.7. Astaxanthin Inhibits ox‐LDL‐Induced Caspase‐3 Activation in Early EPCs

Caspase‐3 is a key effector caspase in the apoptotic pathway. Western blot analysis showed that ox‐LDL (20 μg/mL) treatment significantly increased cleaved caspase‐3 levels (1.83 ± 0.13‐fold vs. control, *p* < 0.01; Figure 6H,I). Pretreatment with 50 μg/mL astaxanthin partially but significantly blocked this upregulation, reducing cleaved caspase‐3 levels to 1.32 ± 0.09‐fold vs. control alone group (*p* < 0.01; Figures 6H,I and S14). This finding suggests that astaxanthin’s antiapoptotic effect is, at least in part, mediated by inhibiting caspase‐3 activation.

### 3.8. Astaxanthin Preserves Early EPC Migration and Tube Formation Capacities Against ox‐LDL‐Induced Impairment

EPC migration and tube formation are critical functional properties for vascular repair. Transwell migration assays showed that ox‐LDL (20 μg/mL) treatment for 24 h significantly reduced the migrating early EPCs number from 107 ± 8 (control) to 58 ± 4 (ox‐LDL alone, *p* < 0.01; Figure 7A,C). Pretreatment with 50 μg/mL astaxanthin significantly preserved migratory capacity, increased the migrating early EPCs number to 109 ± 6 (*p* < 0.01 vs. ox‐LDL alone group; Figure 7A,C; control groups, Figure S7). The effects of astaxanthin on early EPC migration could be reversed Nrf2 siRNA treatment.

**Figure 7:**
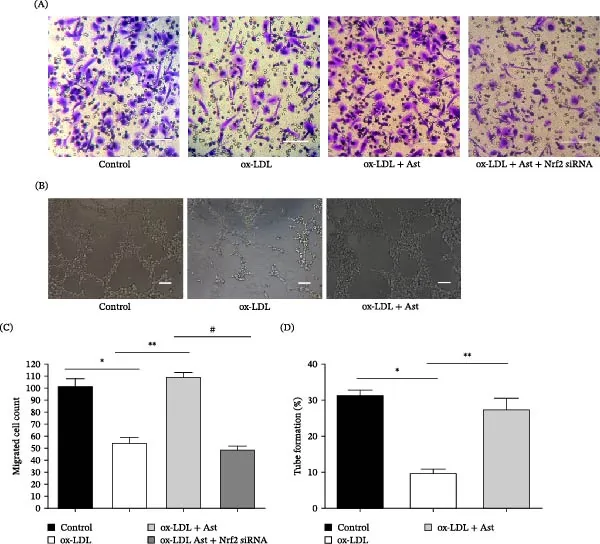
Astaxanthin preserves early EPC migration and tube formation capacities. Early EPCs were pretreated with 50 μg/mL astaxanthin for 6 h, followed by 20 μg/mL ox‐LDL treatment for 24 h (migration assay) or 6 h (tube formation assay). (A, C) Transwell migration assay. ox‐LDL reduced the number of migrated cells, which was reversed by astaxanthin (scale bar: 100 μm). Nrf2 siRNA treatment reduced the effects of astaxanthin on early EPC migration. (B, D) Matrigel tube formation assay. ox‐LDL impaired tube formation (reduced total tube length and branch number), while astaxanthin preserved angiogenic capacity (scale bar: 100 μm). EPC migration and tube formation experiments were performed using samples from six independent donors (*n* = 6). For each donor, migration and tube formation was technically replicated six times, and three image fields were captured per technical replicate. The average number of migrating early EPCs and the average number of tube branches were calculated for each donor and expressed as mean ± SD. These donor‐level values were then used for statistical comparison among groups. Comparisons among multiple groups were performed using one‐way analysis of variance (ANOVA) followed by Tukey’s post hoc test, with SPSS 22.0.  ^∗^ *p* < 0.01 vs. control;  ^∗∗^ *p* < 0.01 vs. ox‐LDL group; #*p* < 0.01 vs. ox‐LDL + astaxanthin group. Ast, astaxanthin.

Tube formation assays on Matrigel showed that ox‐LDL treatment severely impaired early EPCs angiogenic potential, as evidenced by a significant reduction in total tube length (37 ± 4 mm/field vs. 113 ± 9 mm/field in control, *p* < 0.01; Figure 7B,D) and number of tube branches (10 ± 2 vs. 31 ± 2 in control, *p* < 0.01; Figure 7B,D). Pretreatment with 50 μg/mL astaxanthin significantly enhanced tube formation, increased total tube length to 97 ± 8 mm/field and tube branches to 28 ± 3 (*p* < 0.01 vs. ox‐LDL alone group, both; Figure 7B,D). These results demonstrate that astaxanthin effectively mitigates ox‐LDL‐induced functional impairment of early EPCs. Nrf2 siRNA reversed the protective effect of astaxanthin on early EPC migration; however, whether Nrf2 knockdown also reverses the protective effect of astaxanthin on tube formation was not assessed.

## 4. Discussion

Atherosclerotic cardio‐cerebrovascular diseases remain a leading global health burden, with endothelial dysfunction recognized as a key initiating event in their pathogenesis [16]. Endothelial cells form the critical inner lining of blood vessels, maintaining vascular homeostasis through the regulation of vasomotor tone, coagulation, and inflammatory responses [17]. EPCs, derived primarily from bone marrow, serve as endogenous repair cells that migrate to sites of vascular injury, differentiate into mature endothelial cells, and replenish damaged endothelial layers [1]. Their functional integrity—encompassing proliferation, migration, and angiogenic capacity—is therefore essential for vascular repair and the prevention of atherosclerotic progression [18]. However, pathological conditions associated with elevated oxidative stress, such as hyperlipidemia, diabetes, and aging, severely impair the EPC function [19]. In this study, EPCs were cultured from PBMCs and characterized functionally by their proliferative capacity, colony‐forming unit formation, Dil‐ac‐LDL uptake, and UEA‐1 lectin binding, as well as immunophenotypically by the positive expression of CD34, CD133, and VEGFR2/KDR. These cells appeared within 4–7 days of culture, exhibited a spindle‐shaped morphology, and expressed high levels of stem cell surface markers, consistent with early EPCs, as opposed to late outgrowth endothelial cells (OECs), which typically emerge after 2–3 weeks. ox‐LDL, a central mediator of atherosclerotic injury, induces excessive ROS production, disrupts the antioxidant defense system, and triggers EPC senescence and apoptosis [20]—consistent with our findings that ox‐LDL exposure reduced EPC GSH levels, inhibited Nrf2 nuclear translocation, downregulated NQO1 and HO‐1 expression, and impaired EPC migration and tube formation. These results underscore the critical role of EPC protection in targeting atherosclerotic pathogenesis.

Astaxanthin, a natural carotenoid with exceptional antioxidant potency, has emerged as a promising candidate for cardiovascular protection [4]. Synthesized by microalgae such as Haematococcus pluvialis under stress conditions, astaxanthin’s unique chemical structure—featuring two hydroxyl and two ketone groups at its terminal rings—enables it to cross cell membranes efficiently, scavenge a broad spectrum of free radicals, and prevent lipid peroxidation [21]. Unlike conventional antioxidants that merely neutralize ROS, astaxanthin exerts pleiotropic cytoprotective effects, including the upregulation of endogenous antioxidant genes, preservation of mitochondrial function, and induction of autophagy [5, 22, 23]. Mitochondria are both major sources of intracellular ROS and primary targets of oxidative damage; mitochondrial depolarization, as observed in our ox‐LDL‐treated early EPCs, disrupts ATP production and amplifies ROS release, creating a vicious cycle of oxidative stress [24]. Our data demonstrate that astaxanthin pretreatment reversed ox‐LDL‐induced MMP loss, reduced ROS accumulation, and restored GSH levels—highlighting its ability to break this cycle. Notably, astaxanthin’s antioxidant efficacy in early EPCs extends beyond direct ROS scavenging, as evidenced by its induction of Nrf2‐mediated NQO1 and HO‐1 expression, suggesting a dual mechanism of action that enhances both direct and indirect antioxidant defense systems. Future studies could further explore astaxanthin’s effects on mitochondrial biogenesis and respiratory chain function to fully elucidate its mitochondrial protective role in early EPCs.

Nrf2 is widely recognized as the master regulator of cellular antioxidant responses, orchestrating the transcription of ARE‐driven genes involved in ROS detoxification, GSH synthesis, and xenobiotic metabolism [8, 10]. In EPCs, Nrf2 activation is critical for maintaining their regenerative capacity under oxidative stress: previous studies have shown that Nrf2 deficiency exacerbates EPC dysfunction in atherosclerotic models, while Nrf2 activation via pharmacological agents restores EPC survival and angiogenic function [25, 26]. Our study extends these findings by demonstrating that astaxanthin’s protective effects on early EPCs are dependent on Nrf2 activation—supported by the observation that Nrf2 siRNA knockdown abolished astaxanthin‐induced NQO1 and HO‐1 upregulation and ROS reduction. ox‐LDL’s inhibition of Nrf2 nuclear translocation, as observed in our experiments, likely reflects its disruption of Nrf2‐Kelch‐like ECH‐associated protein 1 (Keap1) interaction or promotion of Nrf2 ubiquitination and degradation [27]—a process that astaxanthin may counteract by stabilizing Nrf2 or facilitating its nuclear import. This Nrf2‐dependent mechanism aligns with astaxanthin’s known role in activating antioxidant signaling in other cell types [28, 29] and establishes Nrf2 as a key mediator of its early EPCs‐protective effects.

The PI3K/Akt pathway is a central regulator of cell survival, proliferation, and metabolism, and its activation has been linked to Nrf2‐mediated antioxidant responses [30]. GSK‐3, a downstream target of Akt, functions as a negative regulator of Nrf2 by phosphorylating Nrf2 and promoting its cytoplasmic retention and degradation [31]. Our data show that astaxanthin enhanced Akt phosphorylation (activation) and decreased GSK‐3β (Tyr‐216) phosphorylation, indicating its inactivation, while pretreatment with the PI3K inhibitor LY294002 abrogated astaxanthin‐induced Nrf2 nuclear translocation, NQO1 and HO‐1 upregulation, and GSH restoration. These results confirm that astaxanthin activates Nrf2 via the PI3K/Akt/GSK‐3 pathway—consistent with previous reports in retinal pigment epithelial cells and hepatocytes [32, 33]. This signaling cascade provides a clear molecular framework for astaxanthin’s protective effects: PI3K/Akt activation inhibits GSK‐3, thereby preventing Nrf2 degradation and promoting its nuclear accumulation, which in turn upregulates antioxidant genes and suppresses oxidative stress‐induced apoptosis. Notably, astaxanthin’s inhibition of ox‐LDL‐induced caspase‐3 expression further supports this antiapoptotic mechanism, as caspase‐3 activation is a downstream consequence of mitochondrial dysfunction and oxidative stress [34]. The inclusion of a caspase‐3 inhibitor as a positive control in future studies could strengthen the causal link between caspase‐3 inhibition and astaxanthin’s antiapoptotic effects.

The astaxanthin‐mediated PI3K/Akt/Nrf2 signaling pathway has been reported in several types of cells (such as epithelial cells [35], retinal photoreceptor cells [36], macrophages [37], and medial septal cells [38]), but its role in endothelial cells, particularly early EPCs, has not been fully elucidated. Early EPCs are relatively proliferative progenitor cells that play a crucial role in vascular endothelial repair and angiogenesis in ischemic tissues, such as infarcted myocardium. The ischemic states of these tissues are often accompanied by severe oxidative stress and inflammation [39], placing high demands on the cellular antioxidant capacity. Therefore, understanding the antioxidant mechanism of early EPCs is crucial for improving their viability and repair capacity in these tissues. Astaxanthin is a widely distributed antioxidant in shrimp and crabs, previously understood to have a strong quenching effect on ROS (singlet oxygen and free radicals) [40]. However, this direct antioxidant effect is heavily dependent on its intracellular concentration and distribution. The PI3K/Akt/Nrf2 signaling pathway is a crucial antioxidant pathway in early EPCs. This study found that astaxanthin achieves more sustained antioxidant effects in early EPCs through the activation of this signaling pathway and the subsequent expression of antioxidant genes. This mechanism helps us to understand the relatively lower incidence of cardiovascular disease in coastal areas than in inland areas. Furthermore, Nrf2 exerts its antioxidant effect mainly through its nuclear translocation [41]. In this study, we found that astaxanthin promotes the nuclear translocation of Nrf2 in early EPCs, thereby clarifying the key step of astaxanthin’s antioxidant effect in these cells. These results lay a solid foundation for future investigation on astaxanthin’s antioxidant mechanism in the cardiovascular system and its protective effects against cardiovascular disease.

Several limitations of the present study should be acknowledged. First, our experiments were conducted in vitro using human early EPCs isolated from healthy volunteers, and the translational relevance to atherosclerotic patients—who exhibit complex metabolic disturbances and systemic inflammation—requires validation in animal models and clinical trials. These results do not establish a therapeutic application for atherosclerotic disease prevention or treatment. Second, while we focused on the PI3K/Akt/GSK‐3/Nrf2 pathway, astaxanthin may exert additional protective effects via other signaling pathways (e.g., nuclear factor‐κB [NF‐κB] or mitogen‐activated protein kinases [MAPKs]) that warrant investigation. Third, the optimal concentration and duration of astaxanthin treatment for EPC protection need to be refined in vivo, considering factors such as bioavailability, tissue distribution, and potential interactions with other nutrients or medications.

## 5. Conclusions

In conclusion, our study indicates that astaxanthin protects early EPCs against ox‐LDL‐induced oxidative stress, apoptosis, and functional impairment by activating the PI3K/Akt/GSK‐3 pathway, promoting Nrf2 nuclear translocation, and enhancing the endogenous antioxidant defense system. These results uncover a novel mechanism underlying the cardiovascular protective effects of astaxanthin; however, its clinical application in preventing atherosclerotic cardiovascular and cerebrovascular diseases remains to be established, given the limitations inherent in in vitro data. Therefore, further in vivo and clinical studies are warranted to validate the safety and efficacy of astaxanthin in enhancing the EPC function and vascular health in patients with atherosclerosis.

**Nomenclature**
ARE: Antioxidant response element
DCFH‐DA: Dichloro‐dihydro‐fluorescein diacetate
EPCs: Endothelial progenitor cells
GAPDH: Glyceraldehyde‐3‐phosphate dehydrogenase
GSK‐3: Glycogen synthase kinase 3
JC‐1: 5,5′,6,6′‐tetrachloro‐1,1′,3,3′‐tetraethylbenzimidazolcarbocyanine iodide
MMP: Mitochondrial membrane potential
NQO1: Nicotinamide adenine dinucleotide phosphate (NAD[P]H) quinone oxidoreductase
Nrf2: Nuclear factor‐E2‐related factor 2
PI3K: Phosphoinositide 3‐kinase
ROS: Reactive oxygen species.

## Author Contributions

Writing – review and editing: Qijun Jiang, Keqing Pan, and Xiao Xu. Methodology, operation: Zhigang Gong and Zhigang Li. Conceptualization: Shifang Ding. Supervision: Meichun Zhang.

## Funding

This research was funded by a grant from Youth Fund of the National Science Foundation of China (Grant 81600318) and the Natural Science Foundation of Hubei Province of China (Grant 2023AFB1066).

## Disclosure

All authors have read and agreed to the published version of the manuscript.

## Ethics Statement

The study was conducted in accordance with the Declaration of Helsinki and approved by the Institutional Review Board of the Puren Hospital Affiliated to Wuhan University of Science and Technology (Approval Number 2026018). The inclusion criteria required that all donors be free of hepatitis, HIV, chronic liver or kidney disease, advanced‐stage malignancy, hypertension, diabetes, hyperlipidemia, or cardiovascular disease. To ensure anonymization, all personal identifying information (e.g., names and contact details) was replaced with unique numeric codes upon sample collection, and the data were analyzed in a deidentified manner to protect donor confidentiality.

## Consent

The informed consent for clinic and research usage of the blood sample were obtained from all blood donors involved in the study.

## Conflicts of Interest

The authors declare no conflicts of interest.

## Supporting Information

Additional supporting information can be found online in the Supporting Information section.

## Supporting information


**Supporting Information** Figure S1: Cell Viability of EPCs. EPCs were pretreated with 20 μg/mL ox‐LDL for 24 h, astaxanthin (10, 50, or 100 μg/mL) for 6 h, Nrf2 siRNA for 48 h, 1 μM LY294002 for 30 min or 0.1% DMSO for 24 h. Results are normalized to the control group and expressed as mean ± SD (*n* = 6, donor‐level).  ^∗^ *p* < 0.01 vs. control. Ast, astaxanthin. Figure S2: ROS production in EPCs. EPCs were pretreated with control, astaxanthin (50 μg/mL) for 6 h, 1 μM LY294002 for 30 min, Nrf2 siRNA for 48 h, or 0.1% DMSO for 24 h. Results are normalized to the control group and expressed as mean ± SD (*n* = 6, donor‐level). Ast, astaxanthin. Scale bar: 100 μm. Figure S3: Detection of ROS in EPCs with flow cytometry. EPCs were pretreated with 50 μg/mL astaxanthin for 6 h. Ast, astaxanthin. Figure S4: Mitochondrial membrane potential (ΔΨm) assessed by JC‐1 staining in EPCs. EPCs were pretreated with control, 50 μg/mL astaxanthin for 6 h, 1 μM LY294002 for 30 min, Nrf2 siRNA for 48 h, or 0.1% DMSO for 24 h. Results are normalized to the control group and expressed as mean ± SD (*n* = 6, donor‐level). Ast, astaxanthin. Scale bar: 50 μm. Figure S5: Apoptotic morphology detected by DAPI staining in EPCs. EPCs were pretreated with control, 50 μg/mL astaxanthin for 6 h, 1 μM LY294002 for 30 min, Nrf2 siRNA for 48 h, or 0.1% DMSO for 24 h. Results are normalized to the control group and expressed as mean ± SD (*n* = 6, donor‐level). Ast, astaxanthin. Scale bar: 50 μm. Figure S6: Apoptotic rates analyzed by Annexin V‐FITC/PI with flow cytometry of EPCs. EPCs were pretreated with 50 μg/mL astaxanthin for 6 h. Ast, astaxanthin. Figure S7: Transwell migration assay of EPCs. EPCs were pretreated with control, 50 μg/mL astaxanthin for 6 h. Results are normalized to the control group and expressed as mean ± SD (*n* = 6, donor‐level). Ast, astaxanthin. Scale bar: 100 μm. Figures S8–S14: Original uncropped/full‐length western blot images.

## Data Availability

Data are available from the first author upon request.
