# Supplementary material for: Astaxanthin Inhibits ox‐LDL‐Induced Early Endothelial Progenitor Cells Oxidative Stress and Apoptosis Through Nrf2 Activation
Source: Stem Cells Int. 2026 Oct 26;2026:1729848. doi: 10.1155/sci/1729848 (PMC13711028; doi:10.1155/sci/1729848)
Supplement: Supplementary file 1 [file SCI-2026-1729848-s001.zip › Figure S1-Cell Viability-EPCs.docx]

Figure S1 Cell Viability of EPCs

EPCs were pretreated with 20 μg/mL ox-LDL for 24 hours, astaxanthin (10, 50, or 100 μg/mL) for 6 hours, Nrf2 siRNA for 48 hours, 1 μM LY294002 for 30 minutes or 0.1% DMSO for 24 hours. Results are normalized to the control group and expressed as mean ± SD (n = 6). *P < 0.01 vs. control. Ast, astaxanthin.
