# Supplementary material for: Astaxanthin Inhibits ox‐LDL‐Induced Early Endothelial Progenitor Cells Oxidative Stress and Apoptosis Through Nrf2 Activation
Source: Stem Cells Int. 2026 Oct 26;2026:1729848. doi: 10.1155/sci/1729848 (PMC13711028; doi:10.1155/sci/1729848)
Supplement: Supplementary file 1 [file SCI-2026-1729848-s001.zip › Figure S6 Apoptotic rates of EPCs.docx]

Figure S6 Apoptotic rates analyzed by Annexin V-FITC/PI with flow cytometry of EPCs

EPCs were pretreated with 50 μg/mL astaxanthin for 6 hours. Ast, astaxanthin.
