# Supplementary material for: Astaxanthin Inhibits ox‐LDL‐Induced Early Endothelial Progenitor Cells Oxidative Stress and Apoptosis Through Nrf2 Activation
Source: Stem Cells Int. 2026 Oct 26;2026:1729848. doi: 10.1155/sci/1729848 (PMC13711028; doi:10.1155/sci/1729848)
Supplement: Supplementary file 1 [file SCI-2026-1729848-s001.zip › Figure S7 Transwell migration assay of EPCs.docx]

Figure S7 Transwell migration assay of EPCs

EPCs were pretreated with control, 50 μg/mL astaxanthin for 6 hours. Results are normalized to the control group and expressed as mean ± SD (n = 6). Ast, astaxanthin.
