# Supplementary material for: Astaxanthin Inhibits ox‐LDL‐Induced Early Endothelial Progenitor Cells Oxidative Stress and Apoptosis Through Nrf2 Activation
Source: Stem Cells Int. 2026 Oct 26;2026:1729848. doi: 10.1155/sci/1729848 (PMC13711028; doi:10.1155/sci/1729848)
Supplement: Supplementary file 1 [file SCI-2026-1729848-s001.zip › S11.pdf]

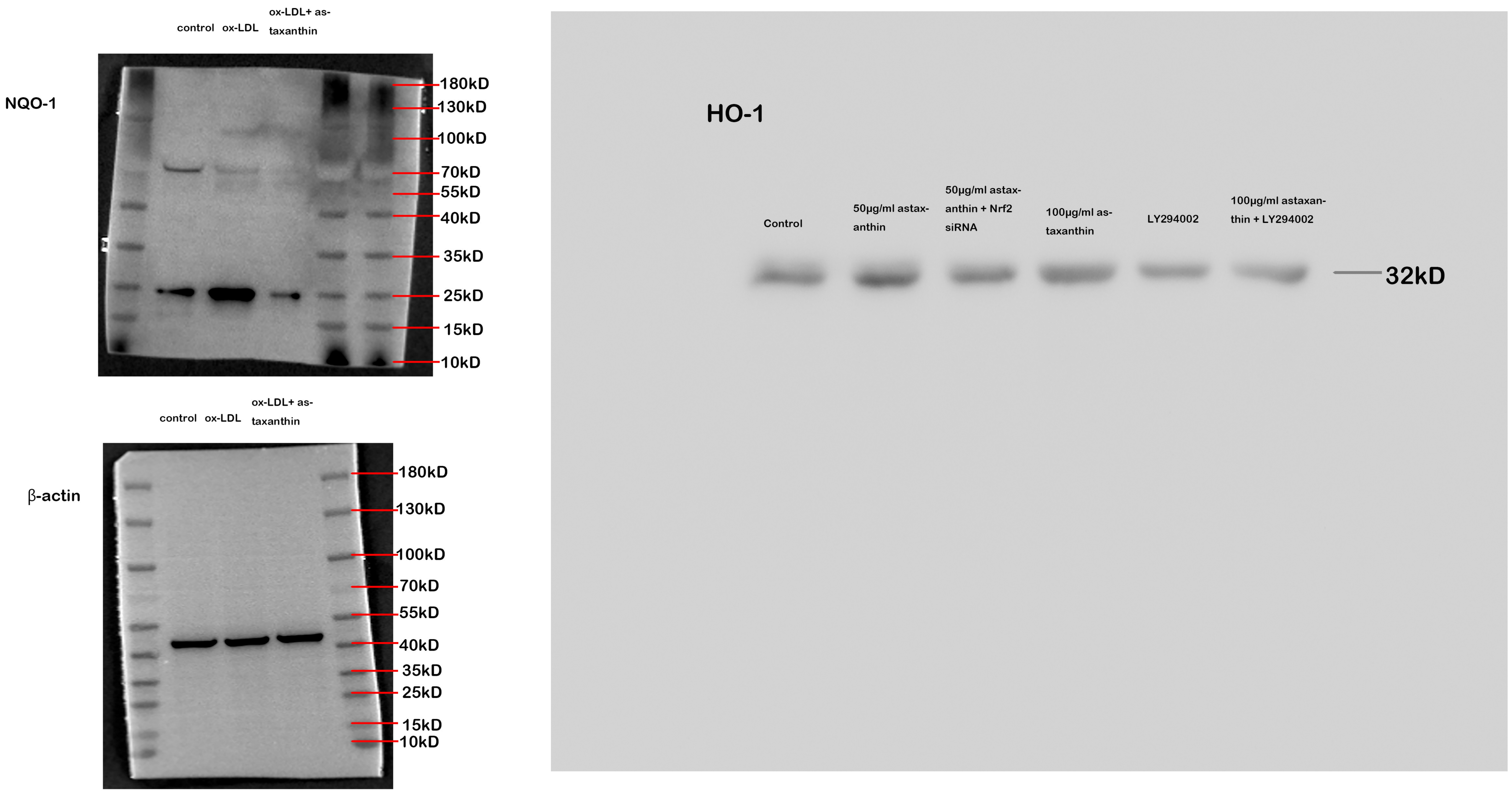
NQO-1
control
ox-LDL
ox-LDL+ as-taxanthin
180kD
130kD
100kD
70kD
55kD
40kD
35kD
25kD
15kD
10kD
β-actin
control
ox-LDL
ox-LDL+ as-taxanthin
180kD
130kD
100kD
70kD
55kD
40kD
35kD
25kD
15kD
10kD
HO-1
Control
50μg/ml astax-anthin
50μg/ml astax-anthin + Nrf2 siRNA
100μg/ml as-taxanthin
LY294002
100μg/ml astaxan-thin + LY294002
32kD
