# Supplementary material for: Astaxanthin Inhibits ox‐LDL‐Induced Early Endothelial Progenitor Cells Oxidative Stress and Apoptosis Through Nrf2 Activation
Source: Stem Cells Int. 2026 Oct 26;2026:1729848. doi: 10.1155/sci/1729848 (PMC13711028; doi:10.1155/sci/1729848)
Supplement: Supplementary file 1 [file SCI-2026-1729848-s001.zip › S12.pdf]

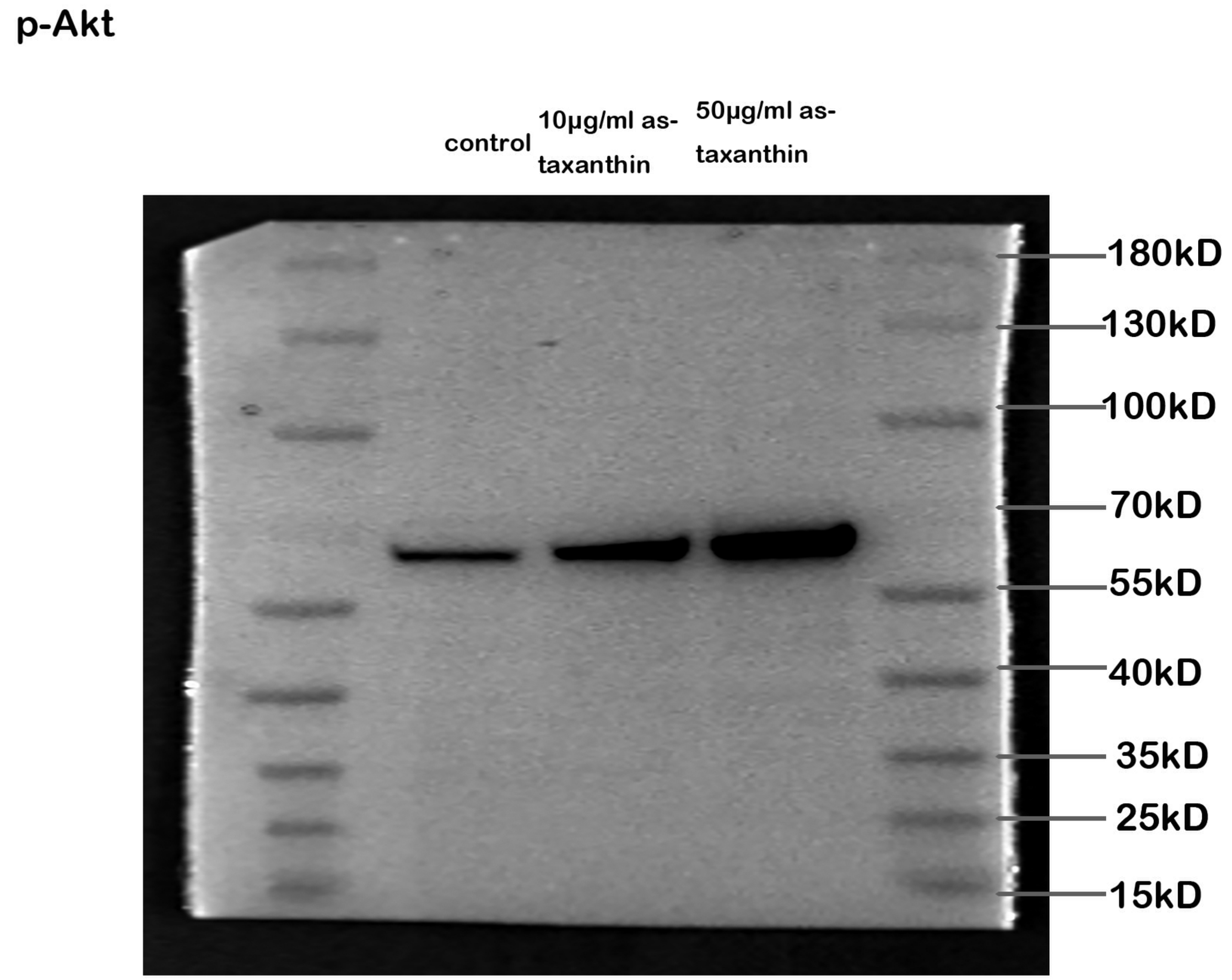
p-Akt
control
10μg/ml as-taxanthin
50μg/ml as-taxanthin
180kD
130kD
100kD
70kD
55kD
40kD
35kD
25kD
15kD

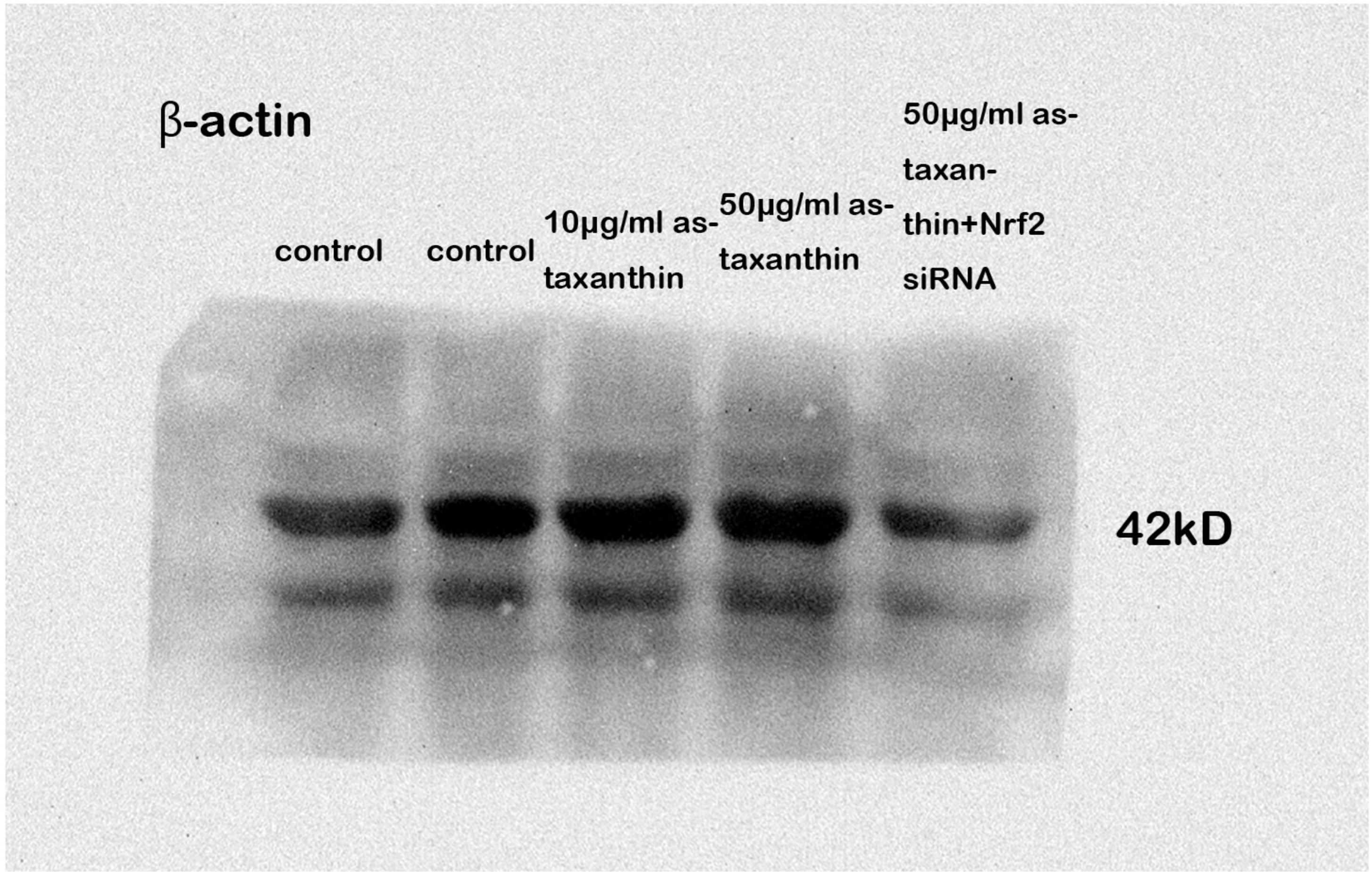
β-actin
control
control
10μg/ml as-taxanthin
50μg/ml as-taxanthin
50μg/ml as-taxan-thin+Nrf2 siRNA
42kD

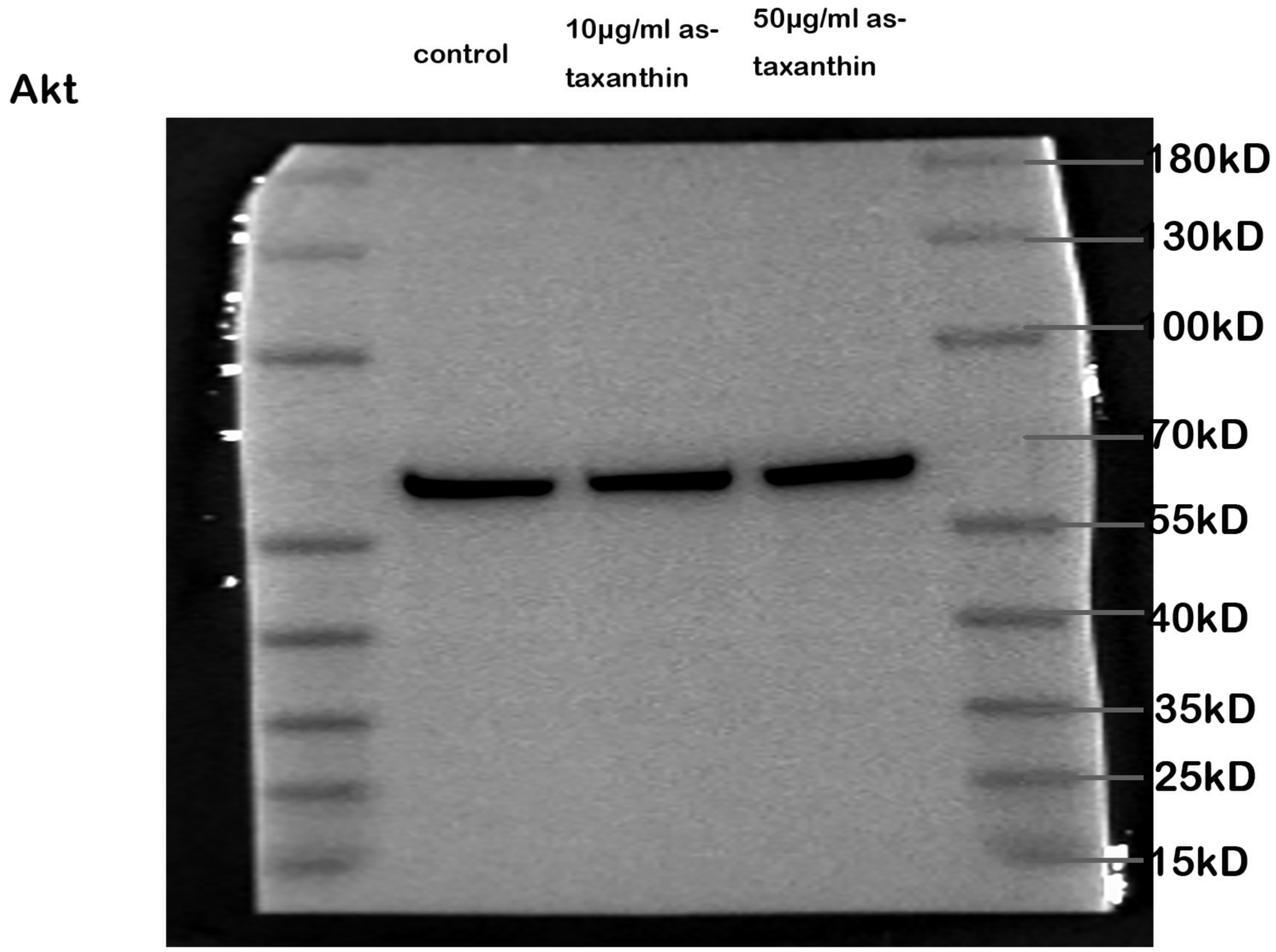
Akt
control
10μg/ml as-taxanthin
50μg/ml as-taxanthin
180kD
130kD
100kD
70kD
55kD
40kD
35kD
25kD
15kD
